# Influences of environmental and leaf functional traits variations on photosynthetic characteristics of *Cotoneaster multiflorus* in Xinglong Mountain

**DOI:** 10.3389/fpls.2025.1562491

**Published:** 2025-08-05

**Authors:** Ling Han, Xiaodong Ma, Chengzhang Zhao, Dingyue Liu

**Affiliations:** ^1^ School of Art and Design, Lanzhou Jiaotong University, Lanzhou, China; ^2^ College of Geography and Environmental Science, Northwest Normal University, Lanzhou, China; ^3^ Xinglongshan Forest Ecosystem National Positioning Observation and Research Station, Gansu Academy of Forestry, Lanzhou, China

**Keywords:** *Cotoneaster multiflorus*, leaf traits, photosynthetic characteristics, chlorophyll fluorescence, slope aspect

## Abstract

**Background and aims:**

Slope aspect affects the redistribution of solar radiation and precipitation, altering habitat conditions such as temperature, water availability, and soil nutrient composition. However, the impact of slope-induced environmental changes on the synergistic relationship between plant photosynthetic characteristics and leaf functional traits remains underexplored.

**Methods:**

Four plots of *Cotoneaster multiflorus* (C. multiflorus) were established on the southern, eastern, western, and northern slopes within the Xinglong Mountain National Nature Reserve. This study investigated variations in leaf functional traits, photosynthetic-fluorescence characteristics, and environmental responses in *C. multiflorus* across different slope aspects by mathematical statistics.

**Results:**

Our study revealed that the southern slope demonstrated maxima in transpiration rate (Tr), coefficient of non-photochemical burst (NPQ), maximum photosynthetic efficiency of photosystem II (Fv/Fm), vein area (LVA), leaf thickness (LT), and stomatal density (SD). The eastern slope exhibited peak values in net photosynthetic rate (Pn), stomatal conductance (Gs), water use efficiency (WUE), and electron transfer rate of photosystem II (ETR). In contrast, the northern slope showed the highest intercellular CO₂ concentration (Ci), coefficient of photochemical burst (qP), actual photosynthetic efficiency of photosystem II (Y(II)), vein density (VD), and leaf area (LA). Photosynthetic-fluorescence characteristics in *C. multiflorus* were significantly correlated with leaf traits, vein traits, and stomatal density, with VD and SD exerting the most pronounced influences. Photosynthetic physiology on southern and western slopes was differentially modulated by temperature and moisture factors, particularly vapor pressure deficit (VPD) and photosynthetically active radiation (PAR), while the eastern slope was primarily governed by moisture and nutrient availability. Northern slope plants experienced co-regulation by temperature, soil nutrients, and moisture, with soil organic carbon (SOC) and total phosphorus (TP) exhibiting dominant effects.

**Conclusions:**

This research underscores slope-specific adaptive mechanisms and key drivers in *C. multiflorus*, informing scientific cultivation practices for shrub communities in arid ecosystems.

## Introduction

In plants, leaf functional traits determine the ability for effective resource utilization physiological regulation (Ahrens et al., 2020; Meng et al., 2022). Photosynthetic traits are essential for assessing carbon assimilation and represent key mechanisms through which plants adapt to environmental fluctuations and engage in ecological functions (Hussain et al., 2021). Chlorophyll fluorescence induction serves as a valuable tool for evaluating the dynamic interaction between photosynthesis and environmental factors (Christ and Hörtensteiner, 2014). Therefore, a combined analysis of photosynthetic and chlorophyll fluorescence parameters can offer deeper insights into electron transport dynamics in plants, facilitating a more comprehensive and intuitive understanding of plant photosynthesis.

Leaf traits represent the coordinated response and adaptive strategies of plants to their environment. As the primary organs responsible for photosynthesis, gas exchange, water transpiration, and nutrient transport, leaves play a critical role in regulating physiological processes and shaping ecological functions. They serve as essential indicators of a plant’s adaptability and competitiveness within ecological systems (Zhao et al., 2016). Vein traits, as integral components of leaf structure, provide both physiological and mechanical support by facilitating nutrient and water transport, reinforcing leaf structure, and protecting against environmental stress (Ye et al., 2021; Ke et al., 2022). This vascular network directly affects photosynthetic efficiency by enhancing the delivery of resources necessary for photosynthesis and transpiration (Ye et al., 2021). Recent research has increasingly focused on the relationship between plants’ leaf traits and photosynthetic performance. Brodribb et al. (2010) demonstrated a synchronized correlation between leaf vein density and stomatal density (SD) across various environmental conditions, highlighting their interconnected role in regulating photosynthesis. Similarly, Song et al. (2015) revealed that increased vein density enhances photosynthetic efficiency in sorghum and *Perilla*. Ye et al. (2021) further showed that a higher density of secondary veins improves hydraulic conductivity, facilitating gas exchange and boosting photosynthetic capacity, suggesting that vein density is a reliable marker of high photosynthetic potential in rice plants. Despite these insights, limited research has explored the combined role of leaf morphology and physiological traits in enhancing plant adaptability to heterogeneous environments. This gap hinders a broader understanding of plant adaptation strategies and how leaf traits contribute to resilience under varying ecological conditions.

The photosynthetic physiological characteristics of plants are influenced not only by leaf morphology but also by environmental factors such as light, temperature, and soil water content (Subedi et al., 2018; Sagun et al., 2021). Light serves as the primary driver of photosynthesis, significantly impacting carbohydrate distribution and biomass accumulation, which, in turn, affect leaf morphology and photosynthetic performance of plants (Zhang et al., 2019). Limited light availability reduces the photosynthetic rate, restricting nutrient absorption and transport, thereby altering the allocation of dry matter within plants. To compensate for low-light conditions, plants allocate more photosynthetic products to leaf expansion, increasing their surface area to capture additional light quanta (Zhang et al., 2024). Light intensity also regulates stomatal behavior, influencing the rate of CO_2_ uptake. Under excessive light conditions, stomata close to minimize water loss, which reduces transpiration but concurrently inhibits photosynthesis. When light intensity surpasses the saturation point, prolonged stomatal closure can lead to structural damage of photosynthetic organs, resulting in photoinhibition and impairment of photosynthetic machinery (Chen et al., 2021; Bachofen et al., 2020; Carter et al., 2020; Baird et al., 2017). Conversely, prolonged exposure to low-light conditions leads to the development of thinner, darker leaves with reduced dark respiration rates and higher photosystem II (PSII) efficiency (Osada, 2021). Water availability, both in soil and air, further modulates photosynthetic activity by influencing stomatal conductance and transpiration. As plants absorb carbon dioxide through stomata, a substantial amount of water is lost via evaporation. Adequate water supply is essential for sustaining normal physiological functions (Chen et al., 2021). Disruptions in water availability, whether through excess or deficiency, can profoundly alter photosynthetic processes, inhibiting growth and development (Li et al., 2021). Subedi et al. (2018) found that variations in individual stomatal size and density may determine the plant’s ability to adapt to different levels of physiological stress. Despite recognition of the role of light and water in shaping photosynthetic traits, the mechanisms by which plants coordinate resource investment across traits to adapt to heterogeneous environments remain insufficiently understood. Further research is needed to elucidate how plants balance trade-offs between light capture, water use, and photosynthetic efficiency under varying environmental conditions.

The slope aspect affects the redistribution of solar radiation and precipitation, altering habitat conditions such as temperature, water availability, and soil nutrients. These environmental changes significantly affect plant growth patterns and species distribution (Wang et al., 2019). In Xinglong Mountain, *Cotoneaster multiflorus* has exhibited a notable expansion from the northern slope to the southern slope, where it has become the dominant species within the shrub-grass ecosystem. This shift is reshaping the structure and function of plant communities in the region (Davis et al., 2021). Resource competition, specifically for light, water, and nutrients, among neighboring plants under varying slope aspects promotes plasticity in vein traits and photosynthetic characteristics, facilitating adaptation during forest succession (Liu et al., 2023). However, the synergistic interaction between leaf traits and photosynthetic responses to small-scale topographic variations remains poorly understood, limiting insights into shrub adaptation strategies in arid and semi-arid environments. To address this knowledge gap, we established four research plots of *C. multiflorus* on the southern, eastern, western, and northern slopes of Xinglong Mountain. This study examined the differentiation of leaf traits and photosynthetic-chlorophyll fluorescence traits across slopes, explored the relationship between leaf morphology and photosynthetic function, and analyzed the environmental plasticity of leaf functional traits. We hypothesized that (1) the photosynthetic and fluorescence characteristics of *C. multiflorus* vary across slopes aspects, and (2) there are differences in the synergistic effects of the physiological characteristics and leaf functional traits of *C. multiflorus* on different slope aspects.

## Materials and methods

### Study site

The study area is situated within the Xinglong Mountain National Positioning Observation and Research Station (103°50’–104°10’ E, 35°38’–35°58’ N) in Gansu Province at an altitude ranging from 1800 to 3670 m. The region experiences a temperate semi-humid continental climate, characterized by windy, dry springs and increased precipitation during summer (Zhang et al., 2024). The average annual temperature ranges from 3°C to 7°C, with annual precipitation of 420–622 mm, primarily occurring from July to September. Annual evaporation averages 918.6 mm, with a relative humidity of approximately 68%. The area receives 2100–2670 h of sunshine annually, and the frost-free period lasts around 86 days. Total annual solar radiation is estimated at 110–130 kcal/cm². The vegetation in the area comprises a composite ecosystem dominated by forest, with coniferous, broad-leaved, and mixed forests interspersed with shrubland and meadows. Shrubland accounts for over 60% of the total forest area in Xinglong Mountain. Key tree species include *Picea wilsonii*, *Populus davidiana*, and *Betula platyphylla*, whereas the understory hosts abundant shrubs and herbs. Dominant shrubs include *C. multiflorus*, *Berberis kansuensis*, *Lonicera ferdinandii*, and *Fargesii nitida*, whereas *Rubia cordifolia*, *Potentilla bifurca*, and *Fragaria orientalis Losinsk* are the prominent herbs (Zhang et al., 2024; Liu D et al., 2024).

*C. multiflorus* is a shrub species, characterized by ovate to broadly ovate leaves (**Supplementary Figure S1**), it demonstrates strong resistance to drought and poor soil conditions. The plant features dense, above-ground branching and an extensive network of intertwined roots, contributing to soil stabilization, sand preservation, and water conservation. It represents a transitional vegetation type, bridging the arid, low-elevation desert ecosystems, and the forested high-altitude regions of Xinglong Mountain (Liu D et al., 2024).

### Experimental design

A relatively isolated hill was selected and divided into four slope aspects based on topographic maps—south, east, west, and north. Five plots, each measuring 20 m × 20 m, were established on each slope. The longitude, latitude, and elevation of each plot were recorded using a handheld GPS device (Trimble, GeoXH 2008, USA). All plots were situated along the same altitude gradient to ensure consistency in elevation. A zigzag sampling method was implemented across each study plot. Five 3 m × 3 m quadrats were systematically established at four corners and the diagonal intersection point of the plot for concurrent assessment of environmental data, photosynthetic-fluorescence parameters and leaf functional traits data of *C. multiflorus*.

### Slope aspect environmental data

From July to September 2022 (9:00-12:00), the photosynthetic active radiation (PAR) was measured in five quadrats per slope using a handheld optical quantum meter (3415F, Walz, USA Plainfield). Additionally, using a handheld temperature and humidity meter (DT-321S, CEM, China Huashengchang), air temperature (Ta, °C) and relative humidity (RH, %) were measured at a height of 1.5 meters in same quadrats. Each measurement was repeated four times within each quadrat. The saturated water vapor pressure difference (VPD, kPa, Equation 1) was calculated using the following formula (Jin et al., 2023):


(1) $$VPD=0.611\times Exp\left(\frac{17.502Ta}{Ta+240.97}\right)\times \left(1-RH\right)$$

Where Ta is air temperature, RH is relative humidity.

Soil sampling was conducted over 14 consecutive days in mid-July 2022. Four 1 m × 1 m × 0.3 m soil sections were randomly excavated in each quadrats. For each section, the ring knife (200cm³) method was used to obtain soil samples in three layers (with an interval of 10cm). Then, soil samples from the same section are mixed, plant roots and other impurities are removed, and placed in pre-weighed and labeled aluminum boxes for immediate weighing to record their fresh weight. The environmental data in the area are presented in **Supplementary Table S1**.

The collected samples were transported to the laboratory, where they were dried in an oven at 105°C for 12 h until a constant weight was achieved. Soil water content was calculated based on the difference between fresh and dry weights. The soil organic carbon (SOC), total phosphorus (TP) and total nitrogen (TN) were determined by element analyzer (Vario EI, Elementar, German, 2015). Soil temperature at each sampling point was measured and monitored using the WET-2-KIT device. The soil physicochemical properties in the area are presented in **Supplementary Table S2**.

### Photosynthetic parameters

During clear and windless mornings from July to September 2022 (9:00-12:00), on each of the five marked water spindle plants in each slope direction, select one branch from the east, west, south, and north directions, ensuring they have similar lengths, diameters, leaf counts, ages, and canopy positions. On each branch, select a mature and healthy leaf of the water cotoneaster to measure the plant’s photosynthetic-fluorescence parameters. Photosynthesis measurements were conducted using the GFS-3000 portable photosynthesis measurement system (Heinz Walz GmbH, Effeltrich, Germany), equipped with an artificial red and blue light source and a PAR level of 1200 μmol·m^-2^·s^-1^. The CO_2_ concentration was maintained at approximately 420 μmol·mol^-1^, with a flow rate set to 750 μmol·s^-1^. RH was regulated between 40% and 50%, whereas the leaf temperature (T_leaf_) was controlled within a range of 15°C–20°C. Measured variables included the net photosynthetic rate (Pn), transpiration rate (Tr), stomatal conductance (Gs), Water use efficiency (WUE) and intercellular CO_2_ concentration (Ci).

### Chlorophyll fluorescence parameters

Following the measurement of photosynthetic parameters, chlorophyll fluorescence parameters were assessed using a modulated IMAGING-PAM chlorophyll fluorescence meter (Heinz Walz GmbH, Effeltrich, Germany). Actinic light intensity was manually set to 1200 μmol·m^-2^;·s^-1^, and leaves were subjected to dark adaptation for 30 min to determine the initial fluorescence yield (F0) and maximum fluorescence yield (Fm). After dark adaptation, the actinic light was applied, and fluorescence induction curves for each chlorophyll fluorescence parameter were recorded. Data, including the nonphotochemical quenching coefficient (NPQ, Equation 4), electron transport rate (ETR, Equation 6), maximum photosynthetic efficiency (Fv/Fm, Equation 2), actual photosynthetic efficiency (Y(II), Equation 5), and photochemical quenching coefficient (qP, Equation 3), were exported directly from the report window. Parameter calculations followed the methods described by Schreiber et al. (1994) and Schreiber (2004):


(2) $$\frac{\text{Fv}}{\text{Fm}}=\left(\text{Fm}-\text{F}0\right)/\text{Fm}$$

(3) qP=(Fm'−Fs)/Fv'=1−(Fs–F0')/(Fm'–F0')

(4) $$\text{NPQ}=\left(\text{Fm}–\text{Fm'}\right)/\text{Fm'}=\text{Fm}/{\text{Fm}}^{'}-1$$

(5) Y(II)=(Fm'−Ft)/Fm'

(6) ETR=0.5×Y(II)×PAR×0.84

Where Fm represents the maximum fluorescence after dark adaptation, whereas F0 denotes the dark fluorescence yield. Under light conditions, Fm’ indicates the maximum fluorescence, and F0’ represents the minimum fluorescence. Fv refers to the variable fluorescence resulting from saturated pulsed light, with Fv’ representing the corresponding value under light exposure. Fs signifies the steady-state fluorescence, and Ft corresponds to real-time fluorescence measurements. The photochemical quenching coefficient, qP, is derived from the “lake model,” and PAR stands for photosynthetically active radiation (Tang, 2022).

### Chlorophyll spectral measurement

After determining chlorophyll fluorescence parameters, the leaf chlorophyll spectrum was measured using the portable CID CI-710 fiber spectrometer (Zealquest Scientific Technology Co., Ltd.). Collect leaf transmittance, absorbance, and reflectance spectra using the SpectraSnap software, and record the spectral curves along with the normalized difference vegetation index (NDVI) values. Record the absorbance (OD) at wavelengths 665 nm, 649 nm, 394 and 470 nm, and calculate the contents of chlorophyll a (Chl a, Equation 7), chlorophyll b (Chl b, Equation 8), and carotenoids (Car, Equation 9) using the following formula. Each measurement is repeated six times, with the average value taken as the content of different types of chlorophyll.


(7) $$\text{Chl}\;a=\frac{(13.95\times OD_{665}-6.88\times OD_{649})\times V}{1000\times S}$$

(8) $$\text{Chl}\;b=\frac{(24.96\times OD_{649}-7.32\times OD_{665})\times V}{1000\times S}$$

(9) $$\text{C}ar=\frac{\left[1000\times OD_{470}-2.05\times (13.93\times OD_{665}-6.88\times OD_{649})-114.8\times (24.96\times OD_{649}-7.32\times OD_{665})\right]\times V}{245\times 1000\times S}$$

In the formula, V represents the total volume of the leaf, S denotes the leaf area of fresh leaves, and chlorophyll is measured in μg·cm^-2^. Detailed chlorophyll characteristics of *C. multiflorus* are presented in **Supplementary Figure S3**.

### Leaf trait measurement

The small branches where the physiological characteristics of the leaves were measured were cut along the epidermis of the mother branch and stored in sealed plastic bags to be brought back to the laboratory. The following measurements were conducted on the leaves. Firstly, the leaf area (LA, cm^2^) was measured using the portable laser leaf area meter (CID, Walz, Camas, WA, USA), and the leaf thickness (LT) was measured by using a vernier caliper with an accuracy of 0.01 mm to avoid the vein part. Take the average values respectively as the values of leaf area and leaf thickness. Leaf area (cm²) was measured using a portable laser leaf area meter (CID, CI-202 Walz, Camas, WA, USA, 2015). Then the leaves were fixed with formalin-acetic acid solution (37% formaldehyde solution, 50% ethanol and 13% glacial acetic acid solution). Vein density (LD) was assessed after chemically cleaning the leaves with ethanol containing 5% NaOH, followed by staining with varnish-solid green (Berlyn and Miksche, 1976). Transparent films were created by immersing the stained leaves in water and photographing them under a stereomicroscope (SMZ168-BL, Motic, Hong Kong, China) at 10× magnification. For each leaf, 10 fields of view were captured. After magnifying 40 times again, take a photo of the pore image and calculate the pore density (SD). Vein diameter (VD) and total vein length were analyzed using Motic ImagesPlus 2.0 software. Vein density (VLA) is expressed as total vein length per unit leaf area (mm/mm²).

### Statistical analysis

Leaf functional traits parameters and the photosynthetic-fluorescence parameters of *C. multiflorus* across different slope aspects were analyzed using ANOVA and multiple comparisons through the least significant difference (LSD) method. Significant difference was *α*=0.05. The above statistical analyses were performed by SPSS 22.0 (IBM SPSS Statistics for Windows, Version 22.0. Armonk, NY, USA) and Excel 2016. Data visualization and mapping were performed using Origin 2023 software. Pearson correlation analysis, including Mantel tests, were employed to evaluate the relationships between leaf characteristics (LT, LA, SD, VLA, VD) and photosynthetic-fluorescence characteristics, and the impact of environmental factors on all leaf characteristics. Among them, leaf functional traits are categorized into three types: leaf traits (LT, LA), vein traits (VLA, VD) and stomatal density. Environmental factors are divided into three categories: temperature factors (PAR, ST), water factors(SWC, VPD), and nutrient factors (SOC, TP, TN). Data analysis and image creation are completed on the website (https://www.chiplot.online/). Redundancy analysis (RDA) using CANOCO software version 5 (Biometrics) was applied to examine the contributions of environmental factors to changes in the all leaf functional traits and photosynthetic-fluorescence characteristics, and the synergistic relationship between leaf functional traits and photosynthetic-fluorescence characteristics.

## Results

### Changes in leaf characteristics of *C. multiflorus* across different slope aspects

**Figure 1:**
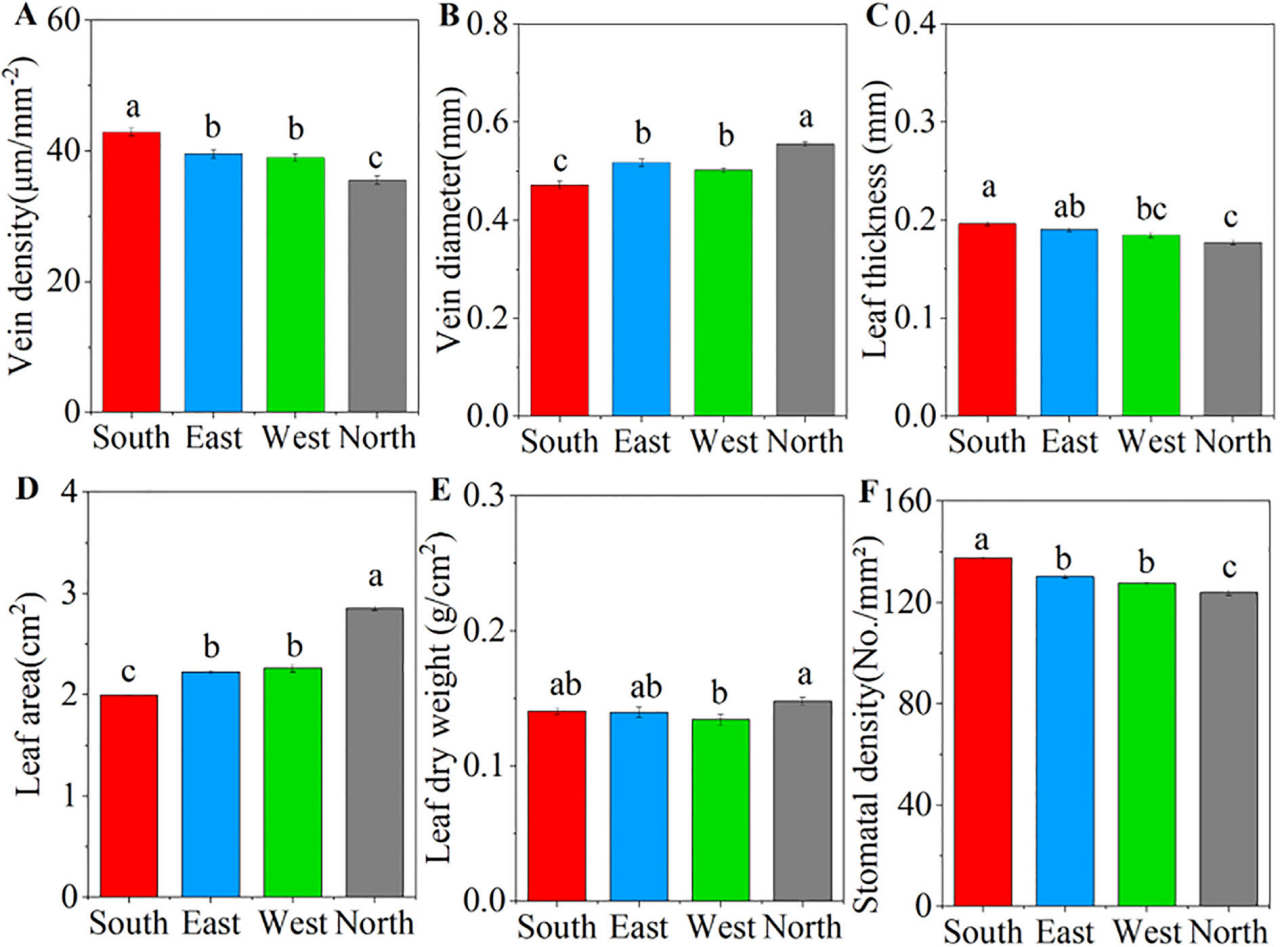
Characteristics of leaf traits of *Cotoneaster multiflorus* under different slope aspects. **(A)** vein density; **(B)** vein diameter; **(C)** leaf thickness; **(D)** leaf area; **(E)** leaf dry weight; **(F)** stomatal density. Different small letters indicate significant differences between groups.

ANOVA results showed that significant differences in the leaf functional traits of *C. multiflorus* were observed across four slope aspects (P< 0.001; **Supplementary Figure S3**). As the slope aspect changes from south, east, and west to north, the vein diameter and leaf area gradually increase. The north slope exhibits a significant increase of 17.67% and 16.32%, respectively, compared to the south slope (P< 0.05; **Figures 1B, D**). In contrast, vein density, leaf thickness, and stomatal density showed a decreasing trend, and the north slope exhibited a substantial reduction of 17.15%, 9.61%, and 9.94% in comparison to the south slope (P< 0.05; **Figures 1A, C, F**).

### Photosynthetic characteristics of *C. multiflorus* under different slope aspects

**Figure 2:**
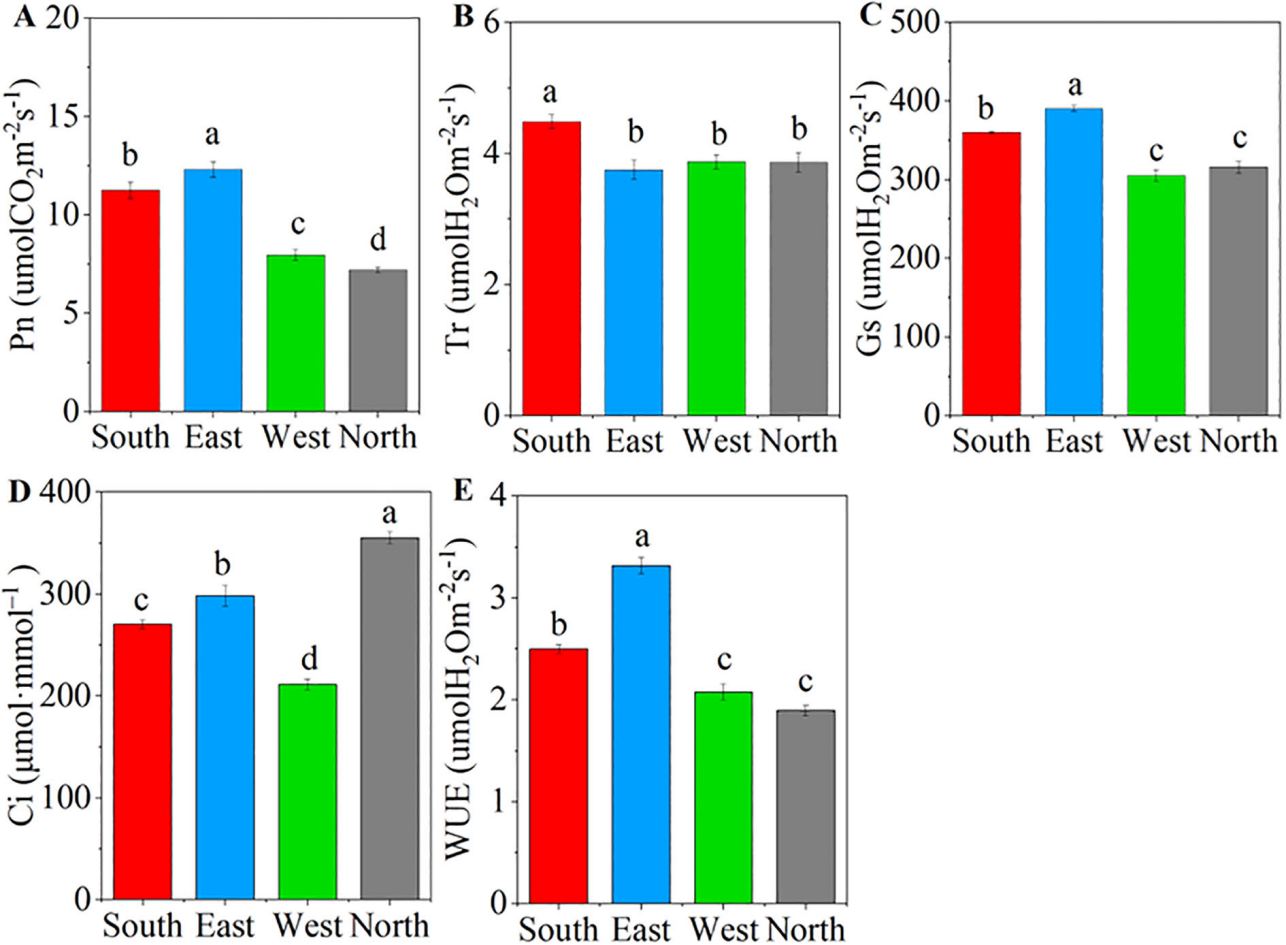
Characteristics of photosynthetic characteristics of *Cotoneaster multiflorus* in different slope aspects. **(A)** photosynthetic rate (Pn); **(B)** transpiration rate (Tr); **(C)** stomatal conductance (Gs); **(D)** intercellular CO_2_ concentration (Ci); **(E)** water use efficiency (WUE). Different small letters indicate significant differences between groups.

ANOVA results showed that significant differences were observed in the photosynthetic parameters of *C. multiflorus* across different slope aspects (P< 0.001; **Supplementary Figure S3**). The Pn, Gs, and WUE increased initially and then declined as the slope aspect shifted from south to north, with the highest values recorded on the eastern slope (**Figures 2A, C, E**). The north slope also decreased by 36.05%, 12.20%, and 24.13% compared to the south slope. The Tr and Ci were significantly greater on the southern and northern slopes, respectively, than on the other slopes (P< 0.05, **Figures 2B, C**).

**Figure 3:**
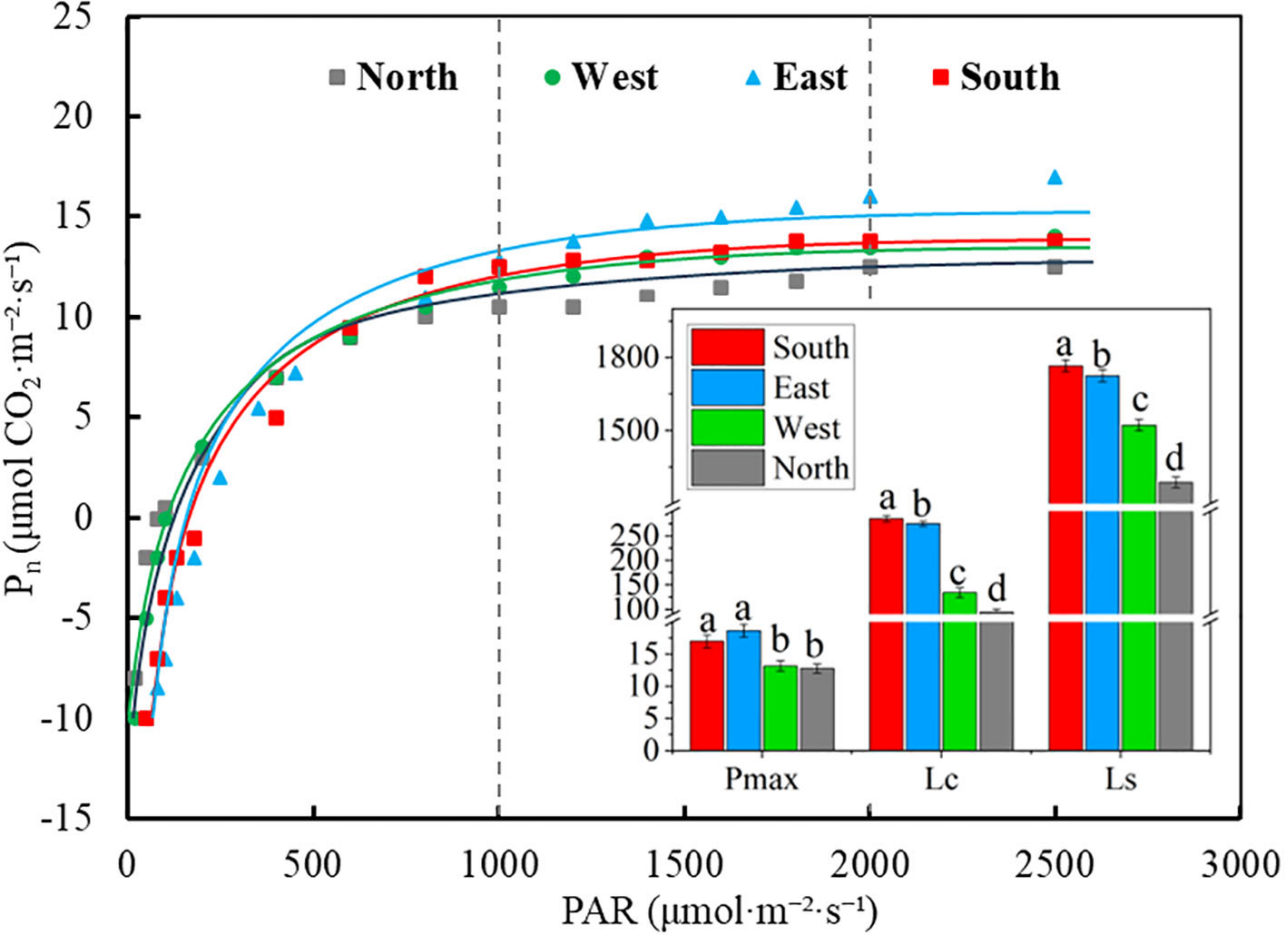
Pn-light response curves of *Cotoneaster multiflorus* with different slope aspects. PAR, photosynthetically active radiation; Pn, photosynthetic rate; Pmax, maximum photosynthetic rate, Lc, light compensation point; Ls, light saturation point.

The light response curve, fitted using the right-angle hyperbolic model (**Figure 3**), revealed significant differences in the maximum net photosynthetic rate, Ls, and Lc of *C. multiflorus* across different slope directions (**Figure 3**; P< 0.05). As the slope aspect changes from south to north, Pmax decreases by 24.91%, reaching its maximum value (18.65 μmol CO_2_·m^-2^·s^-1^) on the eastern slope. Both Lc and Ls exhibited a decreasing trend from the southern to the northern slope, with reductions of 27.05% and 56.28%, respectively (P< 0.05).

### Chlorophyll fluorescence parameters of *C. multiflorus* under different slope aspects

**Figure 4:**
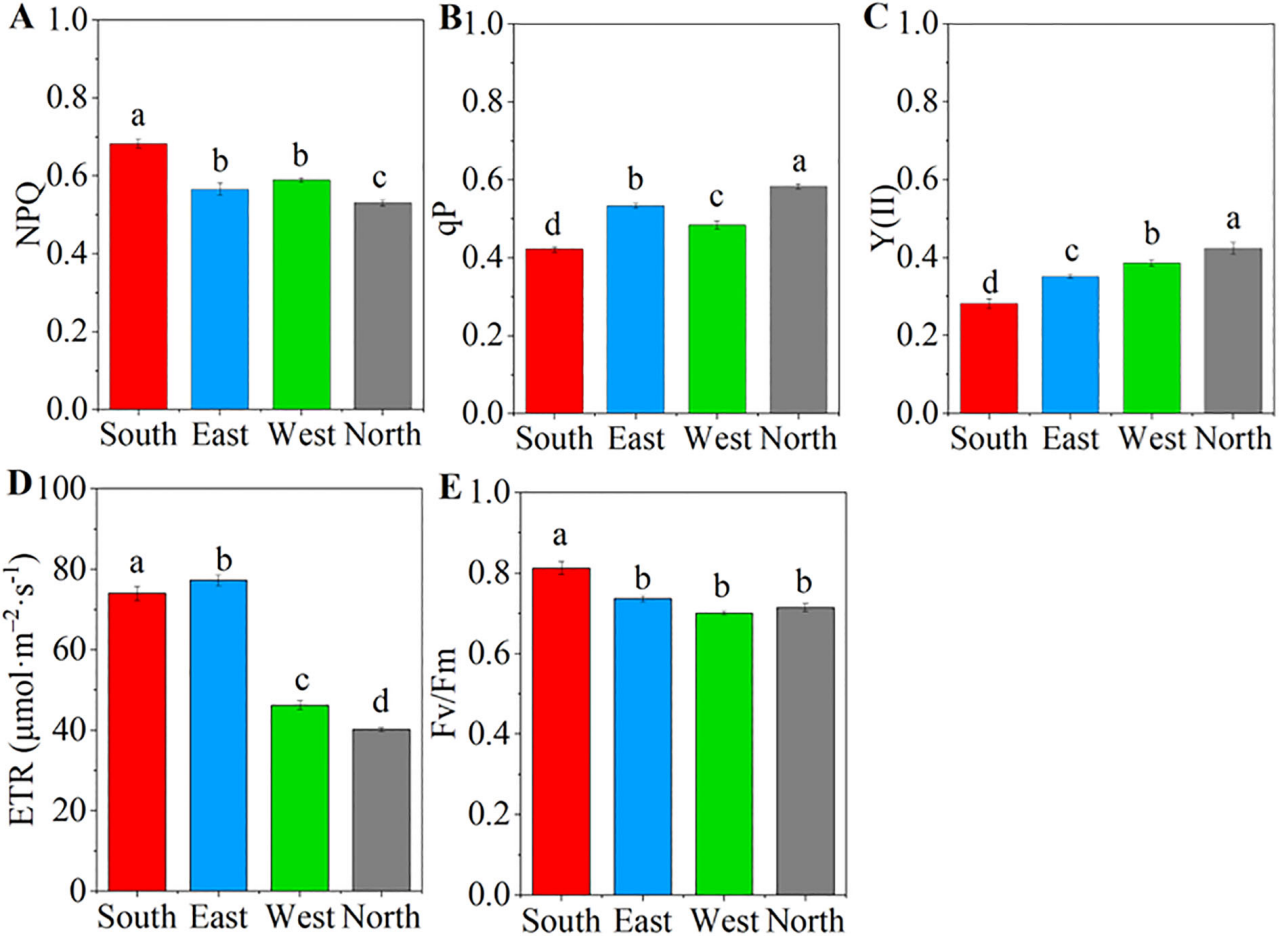
Chlorophyll fluorescence parameters of *Cotoneaster multiflorus* in different slope aspects. **(A)** coefficient of non-photochemical burst (NPQ); **(B)** coefficient of photochemical burst (qP); **(C)** actual photosynthetic efficiency of photosystem II (Y(II)); **(D)** electron transfer rate of photosystem II (ETR); **(E)** maximum photosynthetic efficiency of photosystem II (Fv/Fm). Different small letters indicate significant differences between groups.

ANOVA results showed that significant differences were observed in the chlorophyll fluorescence parameters of *C. multiflorus* across different slope aspects (P< 0.001, **Supplementary Figure S3**). The NPQ showed a downward trend, decreasing by 22.06% from the northern to the southern slope, whereas the qP increased by 34.88% along the same slope variation (P< 0.05; **Figures 4A, B**). The Y(II) exhibited gradual increase trend, rising by 50% from the southern to the northern slope (P< 0.05, **Figure 4C**). The ETR declined after increasing from south to north slope, representing a 45.03% reduction, and the highest value (73.1 μmol·m^-2^·s^-1^) recorded on the eastern slope (P< 0.05, **Figure 4D**). The Fv/Fm was highest on the southern slope and decreased by 13.58% moving toward the northern slope (P< 0.05; **Figure 4E**).

**Figure 5:**
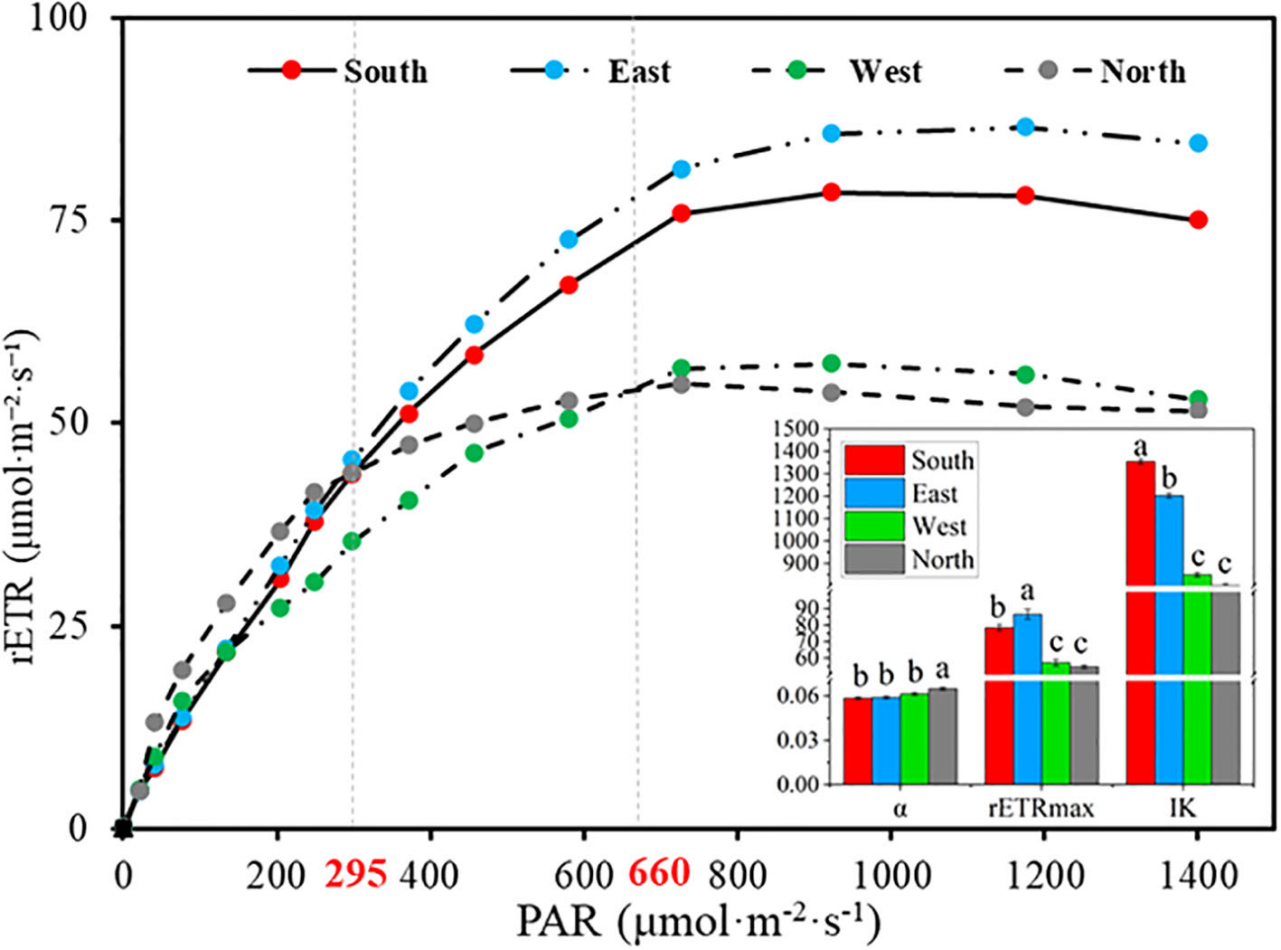
Rapid light-response curve of *Cotoneaster multiflorus* in different slope aspects. rETR, photosynthetic electron transfer rate; PAR, photosynthetically active radiation; α, initial slope; rETRmax, maximum apparent electron transfer rate; IK, half-saturated light intensity.

Rapid light-response curves of *C. multiflorus* across different slope aspects (**Figure 5**) indicated significant differences in the rETR under both low light intensity (<295 μmol·m^-2^·s^-1^) and high light intensity (>295 μmol·m^-2^·s^-1^) (P< 0.05). Under low-light conditions, the rETR in *C. multiflorus* on the northern slope was significantly higher than those on the western, eastern, and southern slopes as PAR increased (**Supplementary Table S4**). Conversely, under high light intensity, the eastern slope exhibited a significantly higher rETR than the southern, western, and northern slopes (**Supplementary Table S4**). Significant differences were also noted in the α, rETRmax, and IK of the rapid light-response curves across slope directions (P< 0.05). As slope aspect shifted from south, east, west to north, α increased by 12.07%, while IK decreased by 40.82% on northern slope. The rETRmax initially increased but subsequently declined, resulting in an overall reduction of 25.09% on northern slope.

### Relationship between the photosynthetic–fluorescence parameters and leaf functional traits in *C. multiflorus*

**Figure 6:**
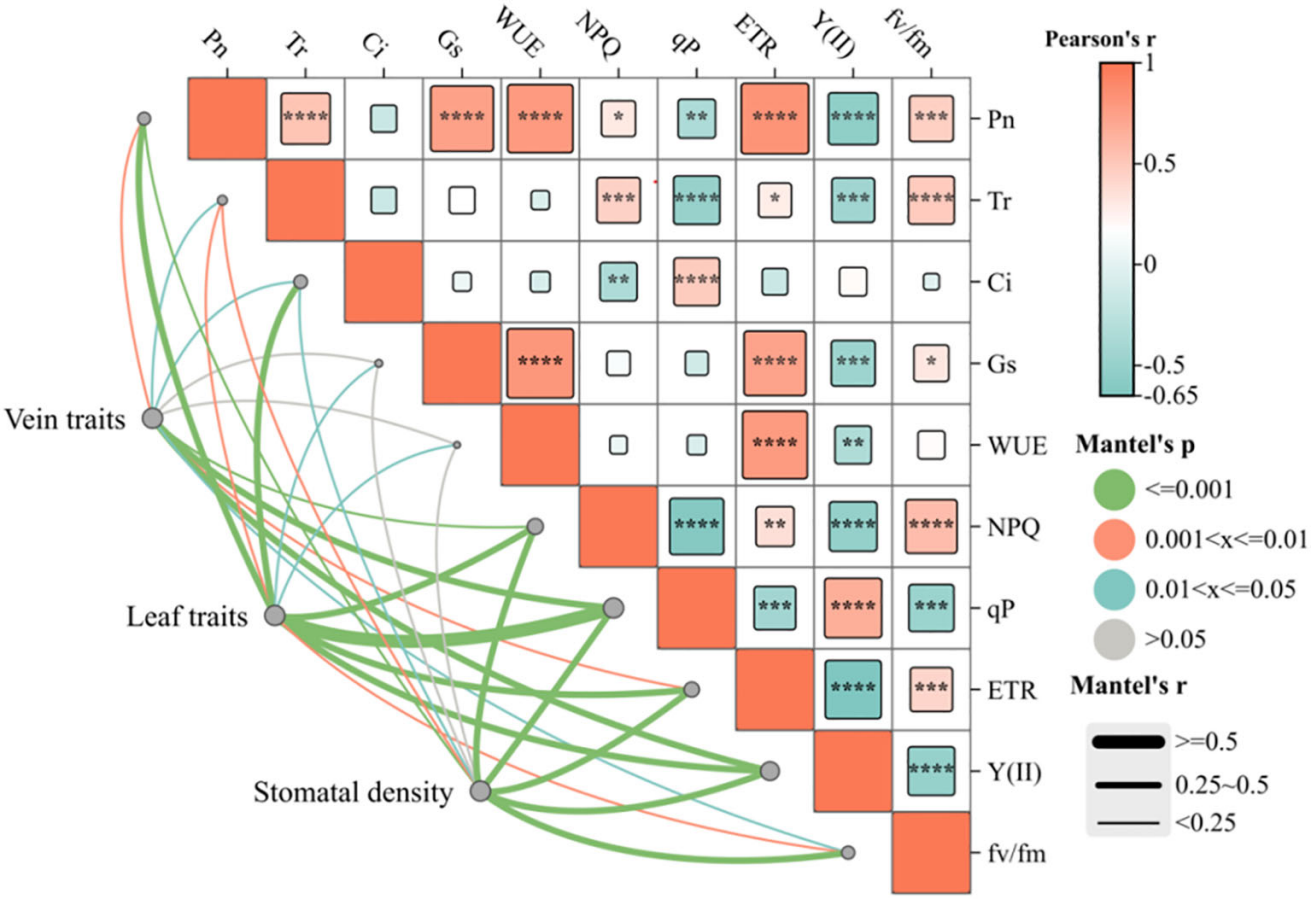
Relationships between leaf traits and photosynthetic–fluorescence characteristics of *Cotoneaster multiflorus*. Photosynthetic parameters: photosynthetic rate (Pn); water use efficiency (WUE); transpiration rate (Tr); intercellular CO_2_ concentration (Ci); stomatal conductance (Gs). Chlorophyll fluorescence parameters: maximum photosynthetic efficiency of photosystem II (Fv/Fm); actual photosynthetic efficiency of photosystem II(Y(II)); electron transfer rate of photosystem II (ETR); coefficient of photochemical burst (qP); coefficient of non-photochemical burst (NPQ). Blue color indicates a negative correlation, and aurantium color indicates a positive correlation. (*P< 0.05, **P< 0.01, ***P< 0.001, ****P<0.0001).

Pearson and Mantel correlation analyses were performed to examine the relationships between leaf characteristics and photosynthetic–fluorescence parameters of *C. multiflorus* (**Figure 6**). Pearson correlation analysis revealed positive correlations of the photosynthetic characteristics (Pn and Tr) and NPQ, ETR and Fv/Fm (P< 0.05), but showed a negative correlation with Y(II) and qP (P< 0.05). Similarly, Gs and WUE was positively correlated with ETR (P< 0.001) and negatively correlated with and Y(II) (P< 0.05). The Mantel test indicated that vein traits(VLA and VD) and stomatal density were extremely significant correlation with Pn, NPQ, qP, ETR, Y(II), and Fv/Fm (P< 0.01), as well as significantly correlated with Ci and Tr (P< 0.05). Leaf traits showed strong correlations with Pn, Ci, NPQ, qP, ETR, Fv/Fm and Y(II) (P< 0.01), as well as significantly correlated with the Tr, Gs and WUE (P< 0.05).

**Figure 7:**
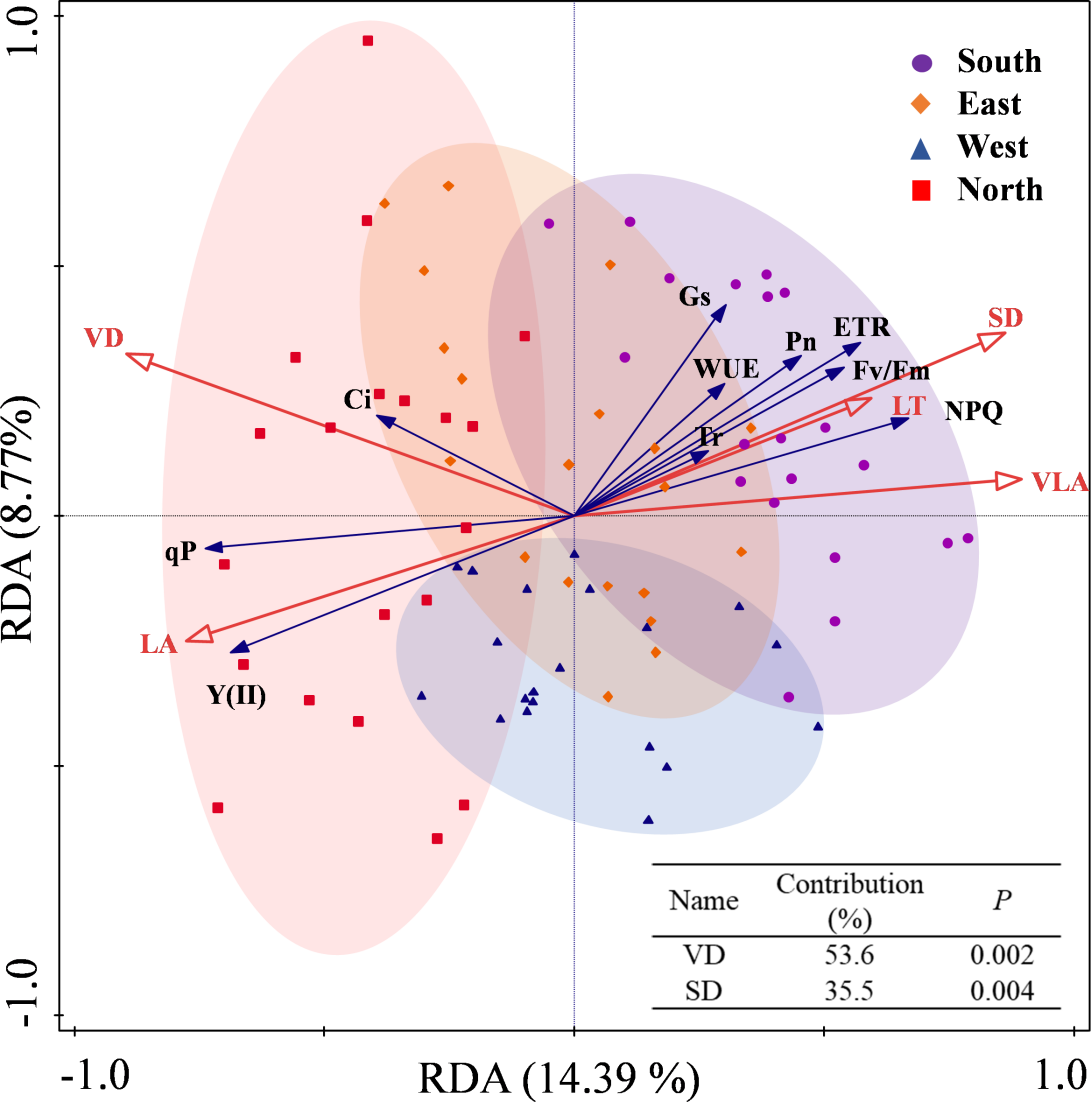
Redundancy analysis for the relation between physiological and leaf functional traits of *Cotoneaster multiflorus* under different slope aspects. VLA, vein density; VD, vein diameter; SD, stomatal density; LT, Leaf thickness; LA, leaf area; Pn, photosynthetic rate; WUE, water use efficiency; Tr, transpiration rate; Ci, intercellular CO_2_ concentration; Gs, stomatal conductance; Fv/Fm, maximum photosynthetic efficiency of photosystem II; Y(II), actual photosynthetic efficiency of photosystem II; ETR, electron transfer rate of photosystem II; qP, coefficient of photochemical burst; NPQ, coefficient of non-photochemical burst.

The redundancy analysis revealed that the first axis explained 14.39% of the variance, whereas the second axis accounted for 8.77%, with the two axes collectively explaining 23.16% of variance in the relationship between leaf traits and photosynthetic–fluorescence parameters (**Figure 7**). VD and SD are the primary factors explaining the variation in the photosynthetic-fluorescence characteristics of *C. multiflorus*, accounting for 89.1% of the contribution (P< 0.01). The photosynthetic-fluorescence characteristics on the north slope and south slope show significant differences. Photosynthetic characteristics (Pn and Gs) and fluorescence characteristics (ETR, Fv/Fm and NPQ) are negatively correlated with VD but positively correlated with SD (P< 0.05). The detailed contributions and explanatory proportions of leaf functional traits to variations in photosynthetic-fluorescence features are presented in **Supplementary Table S5**.

### Relationship between environment factors and physiological parameters of *C. multiflorus*

**Figure 8:**
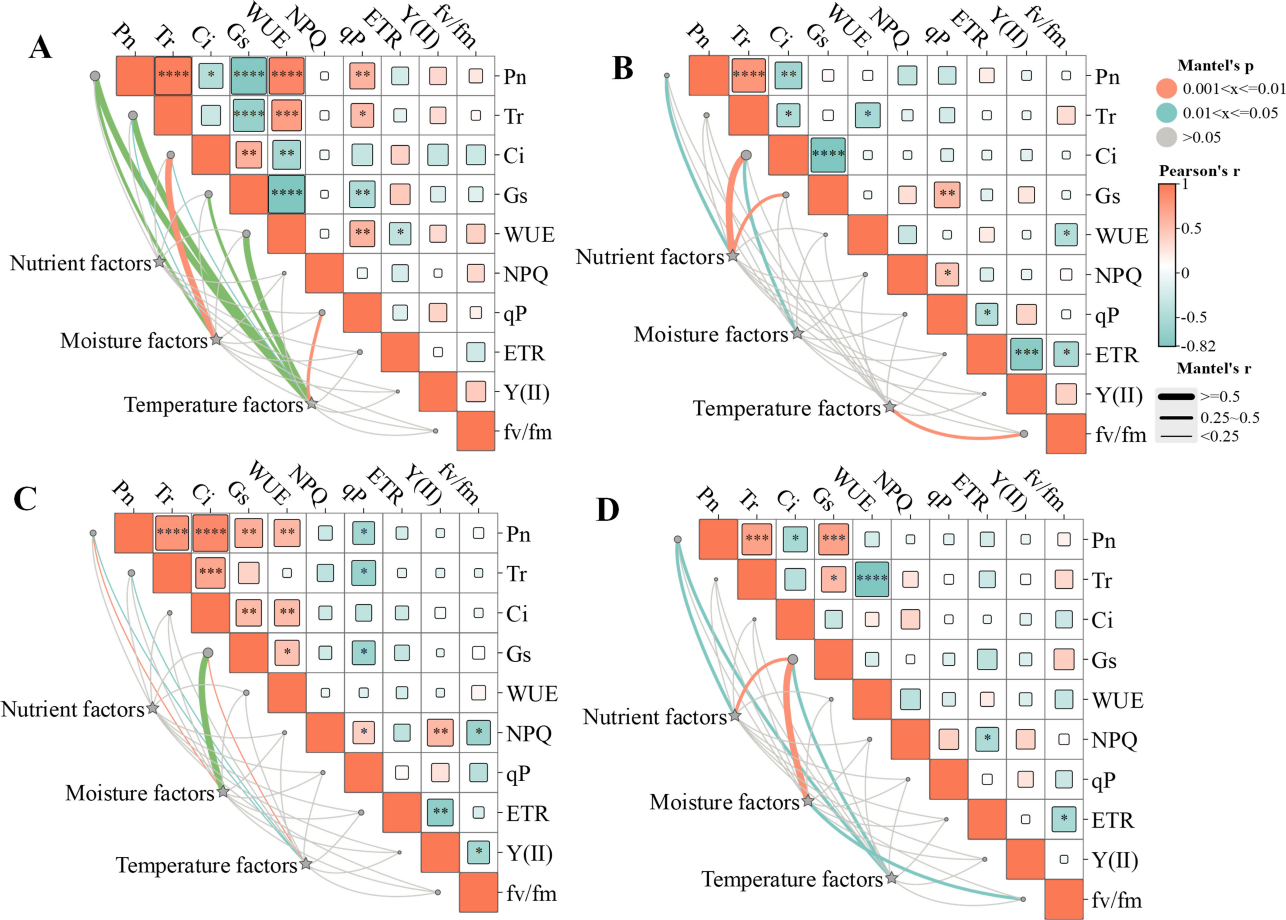
Correlation of environmental factors with photosynthetic–fluorescence characteristics of *Cotoneaster multiflorus* at different slope aspects. **(A)**, southern slope. **(B)**, eastern slope. **(C)**, western slope. **(D)**, northern slope. Pn, photosynthetic rate; WUE, water use efficiency; Tr, transpiration rate; Ci, intercellular CO_2_ concentration; Gs, stomatal conductance. Chlorophyll fluorescence parameters: Fv/Fm, maximum photosynthetic efficiency of photosystem II; Y(II), actual photosynthetic efficiency of photosystem II; ETR, electron transfer rate of photosystem II; qP, coefficient of photochemical burst; NPQ, coefficient of non-photochemical burst; VLA, vein density; VD, vein diameter; SD, stomatal density; LT, Leaf thickness; LA, leaf area. Blue color indicates a negative correlation, and aurantium color indicates a positive correlation. (*P< 0.05, **P< 0.01, ***P< 0.001, ****P<0.0001).

Mantel test results indicated that the influence of environmental factors on the leaf functional traits and physiological characteristics varied by slope direction (**Figure 8**). On the southern slope (**Figure 8A**), temperature factors (PAR and TE) had significant influence on the Pn, Tr, Gs, WUE (P< 0.01) and qP (P< 0.05). Moisture factors (VPD and SWC) also had a significant influence on the Pn and Tr (P< 0.01).On the eastern slope (**Figure 8B**), temperature and moisture factors had significant influence on the Fv/Fm (P< 0.01) and Ci (P< 0.05), respectively. Nutrients factors (SOC, TN and TP) had significant influence on the Pn, Gs and Ci (P< 0.05). On the western slope (**Figure 8C**), Pn and Gs were influenced by temperature and moisture factors (P< 0.05). Similarly, on the northern slope (**Figure 8D**), Pn and Gs were influenced by temperature and moisture factors (P< 0.05), additionally, nutrients factors had significant influence on the Pn and Gs (P< 0.05).

**Figure 9:**
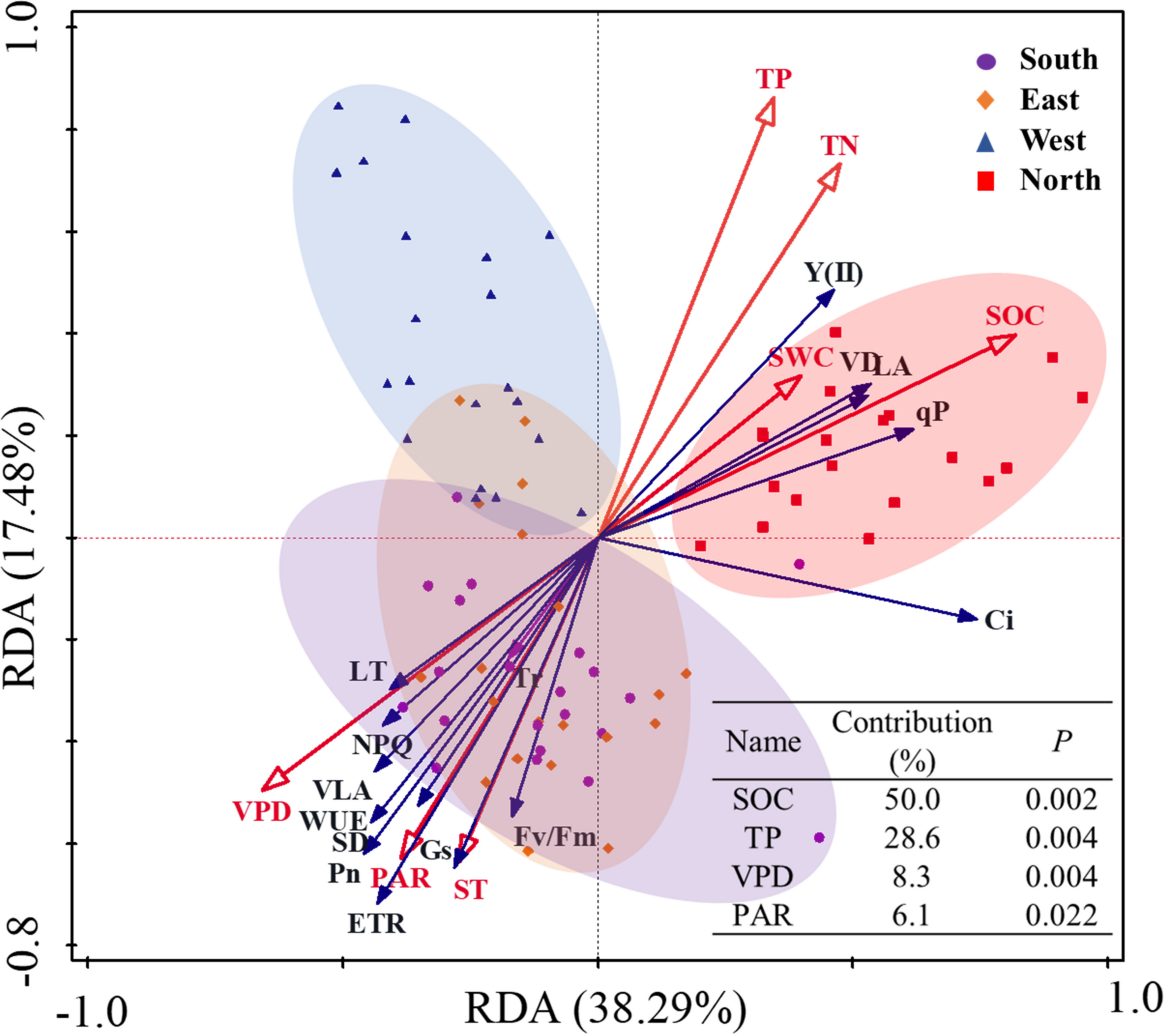
Redundancy analysis of the relation between environmental factors and photosynthetic– fluorescence properties of *Cotoneaster multiflorus* with different slope aspects. VLA, vein density; VD, vein diameter; SD, stomatal density; LT, Leaf thickness; LA, leaf area; Pn, photosynthetic rate; WUE, water use efficiency; Tr, transpiration rate; Ci, intercellular CO_2_ concentration; Gs, stomatal conductance; Fv/Fm, maximum photosynthetic efficiency of photosystem II; Y(II), actual photosynthetic efficiency of photosystem II; ETR, electron transfer rate of photosystem II; qP, coefficient of photochemical burst; NPQ, coefficient of non-photochemical burst; ST, soil temperature; SWC, soil water content; VPD, saturated water vapor pressure difference; SOC, soil organic carbon; TP, total phosphorus; TN, total nitrogen.

RDA was conducted to evaluate the relationships between environmental factors, leaf functional traits, and photosynthetic–fluorescence parameters of *C. multiflorus* under different slope aspects (**Figure 9**). The analysis revealed that the first axis explained 38.29% of the variance, whereas the second axis accounted for 17.48%, collectively explaining 56.77% of variance in the relationship between leaf traits and environmental factors. This suggests that leaf traits were significantly influenced by environmental factors. SOC, TP, VPD and PAR are the primary factors explaining the variation in the leaf functional traits and photosynthetic-fluorescence characteristics of *C. multiflorus*, accounting for 93.0% of the contribution (P< 0.05). Soil factors (SOC and TP) significantly promote Y(II), qP, VD, and LA, while VPD and PAR have a significant positive effect on the remaining leaf functional and physiological traits (P*<* 0.05). A comprehensive breakdown of the detailed contributions and explanatory proportions of all environmental factors affecting leaf traits is provided in **Supplementary Table S6**.

## Discussion

### Environmental and leaf drivers affecting physiological characteristics in *C. multiflorus* on the southern slope

Light is the primary factor affecting plant photosynthesis, and variations in the field light environment profoundly influence plant survival and expansion. Plants exhibit adaptive responses to heterogeneous light conditions (Zhang et al., 2019). Our study reveals that temperature and moisture conditions on southern slope constitute critical environmental factors influencing the photosynthetic-fluorescence characteristics of C. multiflorus (**Figures 8**, **9**). Despite the superior light availability and thermal advantages characteristic of southern slope, the Pn-light response curve demonstrates that the photosynthetically active radiation (PAR) intensity in these habitats frequently exceeds the light saturation point of *C. multiflorus* leaves (**Figure 3**). In response to this photoinhibitory stress, the species exhibits a strategic downregulation of photosynthetic rate (**Figure 2**), likely through the adoption of photoprotective mechanisms. This is consistent with the results of most studies (Jia et al., 2024; Liu ZH et al., 2024), where vegetation under high irradiance conditions typically demonstrates a physiological equilibrium between light capture and utilization, manifesting as reduced photosynthetic activity coupled with enhanced capacity for non-photochemical energy dissipation. The augmentation of carotenoids within photosynthetic pigments has been demonstrated to enhance photoprotective capacity (**Supplementary Figure S3**), as light-harvesting pigment-protein complexes activate thermal energy dissipation mechanisms to mitigate photo-oxidative damage (Sun et al., 2010). Furthermore, elevated values of both maximum photochemical efficiency (Fv/Fm) and non-photochemical quenching coefficient (NPQ) (**Figure 4**) indicate that leaf tissues preferentially dissipate excess energy from photosynthetic electron transport through harmless thermal dissipation via PSII antennae systems (Shafiq et al., 2020). This photoprotective strategy effectively prevents the occurrence of light-induced damage to photosynthetic apparatus by converting surplus excitation energy into heat before it can generate reactive oxygen species.

Simultaneously, intraspecific variation and coordinated shifts in plant functional traits may facilitate vegetation adaptation to heterogeneous and dynamic habitats (Li et al., 2024). Our study revealed significant correlations between photosynthetic-fluorescence characteristics and leaf functional traits in *C. multiflorus* (**Supplementary Figure S2**). Specifically, Mantel test analysis demonstrated that leaf vein traits exerted significant effects on photosynthetic-fluorescence parameters (**Figure 6**). This phenomenon may be attributed to the fact that plants resource investment in venation architecture supports both carbon assimilation and photonic energy management. The coordinated development of vascular structures potentially optimizes the trade-off between photosynthetic efficiency and photoprotective mechanisms, thereby enhancing environmental adaptability. Vein serving as the internal transport network of leaves, higher vein density (**Figure 1A**) facilitates greater water and CO_2_ transport capacity, thereby positively correlating with elevated photosynthetic rates (Ye et al., 2021). In southern slope characterized by reduced soil moisture availability (Table. S2), *C. multiflorus* demonstrates adaptive morphological adjustments to mitigate hydraulic dysfunction and dehydration risks under water stress conditions. Specifically, this species enhances vein density to diminish hydraulic resistance during transpiration, while concurrently optimizing the delivery efficiency of limited water and nutrients for photosynthetic processes (Trueba et al., 2019). This structural modification promotes more efficient matter and energy exchange at the leaf-atmosphere interface. Notably, increased vein density is physiologically associated with greater vein cell proliferation. These additional vascular cells provide augmented pathways for photon distribution and utilization within leaf tissues. The enhanced vascular architecture enables more effective dissipation of excess light energy through non-photochemical quenching (NPQ) mechanisms while simultaneously improving photonic energy conversion efficiency (Wu et al., 2022). Concurrently, increased leaf thickness functions as a compensatory mechanism to sustain photosynthetic water supply by reducing embolism formation risks in mesophyll cells, thereby preserving elevated transpiration rates under vapor pressure deficits (**Figure 2**). Furthermore, *C. multiflorus* specimens from southern slope exhibit elevated stomatal density (**Figure 1F**), an adaptive trait that enhances photosynthetic responsiveness to fluctuating irradiance at the cost of increased water loss propensity, ultimately reducing whole-leaf water-use efficiency (**Figure 2**). These coordinated structural and physiological adjustments have been recognized as essential components of plant photoprotective strategies under combined light and drought stresses (Zhang et al., 2021). These findings collectively demonstrate that leaf physiological and morphological trade-offs occur on south-facing slopes, aligning with Letts et al. (2012) observations of *Buxus sinica* maintaining embolism resistance under low soil water potentials. The allocation of biomass gain to non-assimilatory tissues compromises photosynthetic capacity, representing a fundamental trade-off in concurrent adaptation to irradiance and drought stressors.

### Environmental and leaf drivers affecting physiological characteristics in *C. multiflorus* on the northern slope

Under shaded conditions, plants reduce resource expenditure through leaf morphological adjustments to ensure photosynthetic benefits and enhance survival probability, thereby improving environmental fitness (Poorter et al., 2019). Our investigation reveals that the photochemical characteristics of *C. multiflorus* on northern slope are significantly influenced by light factors (**Figures 8, 9**). This species exhibits minimal photosynthetic rates (Pn, Tr, Gs) and energy transfer efficiency (**Figures 2**, **4**), likely attributable to substantially reduced photosynthetically active radiation limiting photosynthesis, while lower ambient temperature diminishes leaf transpiration through decreased evaporative demand (Flexas and Carriquí, 2020). Consistent with prior research (Yang et al., 2019), we confirm soil nutrient availability as a critical determinant of plant functional traits (**Figures 8**, **9**). Despite reduced photosynthetically active radiation on northern slope, elevated soil moisture and carbon-nitrogen-phosphorus content provide sufficient nutrients for plant development (Feng et al., 2024), prompting *C. multiflorus* to adopt a resource-acquisitive strategy featuring elevated chlorophyll activity and enhanced quantum yield of PSII photochemistry (Y(II)) for low-light adaptation. Concurrently, *C. multiflorus* adapts to light-limited stress by reducing the light compensation point while enhancing PSII photochemical activity (exhibiting the highest photochemical quenching coefficient, qP), thereby accelerating photosynthate production. This strategy aligns with the low-light acclimation mechanism reported in *Dicranopteris pedata* (Liao et al., 2023). Additionally, diminished chlorophyll content (**Supplementary Figure S3**) impairs photon capture and energy transfer, causing significantly reduced net photosynthetic rates in north-slope specimens (Shafiq et al., 2020). However, the decreased Chl a/b ratio indicates enhanced diffuse-light utilization (predominantly blue-violet shortwave photons), representing a critical adaptive benefit for optimizing scattered radiation capture in low-irradiance environments. This finding aligns with the consensus among researchers (Gu et al., 2023; Carter et al., 2020) that reduced Chl a/b ratios represent a characteristic adaptation to shaded conditions.

Our investigation further reveals significant coordination between leaf functional traits and photochemical characteristics in Cotoneaster multiflorus on north-facing slopes, likely facilitated through increased leaf area and enlarged vein diameter (**Figure 1B**) to maintain stable photosynthetic rates and photon conversion efficiency. Broader vein diameter provide greater leaf surface area for photon absorption and utilization (Zhao et al., 2016; Li et al., 2018). Upon photon absorption, light-use efficiency in photosynthesis is enhanced, resulting in elevated quantum yield of PSII photochemistry (Y(II)) (Shi et al., 2022). Additionally, increased vein diameter enables protruding vascular architecture to locally minimize diffuse light reflectance, facilitating full lamina expansion and enhanced photon capture that promotes photochemical quenching and sustains photosynthetic efficiency (Chen et al., 2021). Enlarged vein diameter further transport substantial hydraulic flow and nutrient fluxes (C, N, P) to meet photosynthetic water and elemental demands (Osada, 2021; Leng et al., 2023).

### Environmental and leaf drivers affecting physiological characteristics in *C. multiflorus* on the eastern and western slopes

The allocation of photosynthetic assimilates in plants is co-regulated by intrinsic traits and environmental drivers such as irradiance and soil moisture (Bertolino et al., 2019; Sagun et al., 2021). Our findings indicate sustained high photosynthetic rates on eastern slope, likely attributable to irradiance intensity and duration intermediate between southern and northern slope (**Supplementary Table S1**), establishing a distinct photoperiodic pattern characterized by morning direct radiation and midday diffuse-light dominance. This regime circumvents chronic photoinhibition risks on southern slope and light limitation constraints on northern aspect (**Supplementary Table S1**). Concurrently, significantly higher soil water content relative to southern slope exposures reduces hydraulic constraints, while superior nutrient availability (particularly SOC and phosphorus, **Supplementary Table S2**) exceeds that of intensively leached south slopes and slowly mineralized north slopes (Feng et al., 2024), providing foundational resources for photosynthetic carbon assimilation and energy conversion. Analysis of photosynthetic light-response curves reveals higher light saturation points on eastern slope, likely resulting from a small-thick leaf construction pattern that simultaneously reduces direct photosynthetically active radiation (PAR) interception and enhances photoprotective capacity against high irradiance. Concurrently, elevated stomatal density contributes to superior leaf-level water use efficiency (Subedi et al., 2018). In contrast, the photosynthetic-fluorescence characteristics of plants on the western slope exhibited reductions, attributable to significantly diminished photosynthetically active radiation (PAR), which constrained photosynthetic activity. Lower chlorophyll content (**Supplementary Figure S3**) concurrently compromised light energy conversion efficiency, while reduced environmental temperatures decreased leaf transpiration rates (Osada, 2021).

In contrast, the western slope exhibited reductions in plant photosynthetic-fluorescence characteristics, attributable to significantly diminished photosynthetically active radiation (PAR) constraining photosynthetic activity, lower chlorophyll content compromising light energy conversion efficiency, and decreased environmental temperatures reducing leaf transpiration rates (Osada, 2021). Concurrently, both slopes possessed elevated temperature and soil moisture, forming a unique hydrothermal regime favorable for *C. multiflorus* growth. This species developed moderate leaf morphological structures, allocating leaf biomass to defense structures or increased mesophyll cell density to enhance water use efficiency under limited moisture conditions (Poorter et al., 2019). Furthermore, it coupled enlarged leaf area with fluorescence parameters (e.g., qP, ETR, **Figure 4**), enhancing light availability through increased light-harvesting area to optimize light energy utilization efficiency, thereby facilitating photosynthetic performance (Ye et al., 2021). Consequently, plants on eastern and western slopes potentially exhibit more plastic photosynthetic regulation mechanisms, enabling dynamic adjustments in chlorophyll fluorescence in response to irradiance fluctuations to acclimate to divergent light regimes (Tabassum et al., 2016). Compared to the photoinhibition on southern slopes and light limitation stress on northern slopes, intermittent high-light exposure on east-west slopes may induce a distinctive light protection-utilization trade-off strategy. This is characterized by non-photochemical quenching (NPQ) capacity intermediate between northern (lower) and southern (higher) slopes, while maintaining relatively higher PSII activity. Such an “intermediate strategy” likely confers competitive advantages in heterogeneous habitats, particularly under intensifying global light environment fragmentation, meriting further investigation into these adaptive mechanisms.

## Conclusion

This study elucidates the divergence in photochemical characteristics of *C. multiflorus* across slope aspects and their environmental drivers, revealing the critical role of leaf morpho-physiological integration in adaptive optimization. Confronting high-radiation and drought stress on southern slope, *C. multiflorus* employs a dual-optimization strategy through small-leaf sclerophylly with elevated vein density: (1) enhanced non-photochemical quenching (NPQ) capacity to dissipate excess energy, coupled with (2) accelerated electron transport rates (ETR) ensuring sustained light-harvesting efficiency. This coordinated adjustment effectively balances photoprotective demands with carbon assimilation requirements under combined light and hydraulic constraints. Under the diffuse light and high-humidity conditions characteristic of northern slope, enlarged vein diameter facilitate expansive leaf development that maximizes photon interception. This morphological adaptation, coupled with sufficient soil nutrient availability, optimizes carbon assimilation efficiency in low-irradiance environments. Under the intermediate light-water-nutrient gradient characteristic of eastern and western slope, *C. multiflorus* has evolved highly plastic photosynthetic regulation strategies. The unique diurnal light regime on eastern slope—characterized by morning high-direct radiation transitioning to midday diffuse-light dominance—synergizes with favorable edaphic conditions to drive morpho-physiological integration in *C. multiflorus*. This adaptive optimization concurrently maximizes net photosynthetic rate (Pn), electron transport rate (ETR), and water-use efficiency (WUE), conferring competitive superiority in heterogeneous habitats. On western slope, despite afternoon radiative attenuation, chlorophyll stoichiometric adjustment and dynamic stomatal regulation sustain intermediate photochemical performance. This slope-aspect specific adaptive differentiation exemplifies the evolutionary resource allocation principle: defensive investments dominate in resource-impoverished habitats (e.g., southern slope) versus photosynthetic optimization in resource-rich niches (northern slope). Our findings establish a critical framework for habitat-specific management of plant adaptive phenotypes under global change scenarios, while simultaneously informing physiological design principles for vegetation restoration in degraded montane ecosystems—particularly through strategic matching of ecotypes to microhabitat light-nutrient matrices.

## Data Availability

The raw data supporting the conclusions of this article will be made available by the authors, without undue reservation.
