# Correction: Influences of environmental and leaf functional traits variations on photosynthetic characteristics of *Cotoneaster multiflorus* in Xinglong Mountain

**DOI:** 10.3389/fpls.2025.1702321

**Published:** 2025-10-08

**Authors:** Ling Han, Xiaodong Ma, Chengzhang Zhao, Dingyue Liu

**Affiliations:** ^1^ School of Art and Design, Lanzhou Jiaotong University, Lanzhou, China; ^2^ College of Geography and Environmental Science, Northwest Normal University, Lanzhou, China; ^3^ Xinglongshan Forest Ecosystem National Positioning Observation and Research Station, Gansu Academy of Forestry, Lanzhou, China

**Keywords:** *Cotoneaster multiflorus*, leaf traits, photosynthetic characteristics, chlorophyll fluorescence, slope aspect

“1 School of Art and Design, Lanzhou Jiao tong University, Lanzhou,China

2 College of Geography and Environmental Science, Northwest Normal University, Lanzhou,China

3 Xinglongshan Forest Ecosystem National Positioning Observation and Research Station, Gansu Academy of Forestry, Lanzhou,China”

was erroneously given as

“1 College of Geography and Environmental Science, Northwest Normal University, Lanzhou,China

2 School of Art and Design, Lanzhou Jiao tong University, Lanzhou,China

3 Xinglongshan Forest Ecosystem National Positioning Observation and Research Station, Gansu Academy of Forestry, Lanzhou,China”.

The order of authors in the author list of the published paper was erroneously given as:

“Xiaodong Ma^1^ Ling Han^2*^ Chengzhang Zhao^1,3*^Dingyue Liu^1^”

The correct author list reads:

“Ling Han^1^, Xiaodong Ma^2^, Chengzhang Zhao^2,3*^, Dingyue Liu^2^”

The **Author Contributions** statement was erroneously given as “XM: Conceptualization, Investigation, Writing – original draft. LH: Conceptualization, Funding acquisition, Resources, Supervision, Validation, Writing – review & editing, Writing – original draft. CZ: Conceptualization, Funding acquisition, Project administration, Resources, Supervision, Writing – review & editing. DL: Conceptualization, Investigation, Writing – review & editing”.

The correct **Author Contributions** statement is “LH: Conceptualization, Funding acquisition, Resources, Supervision, Validation, Writing – review & editing, Writing – original draft. XM: Conceptualization, Investigation, Writing – original draft. CZ: Conceptualization, Funding acquisition, Project administration, Resources, Supervision, Writing – review & editing. DL: Conceptualization, Investigation, Writing – review & editing”.

Author “hling0327@163.com, zhaocz601@163.com” was erroneously assigned as corresponding author. The correct corresponding author is “zhaocz601@163.com”.

The original version of this article has been updated.

