# Supplementary material for: Influences of environmental and leaf functional traits variations on photosynthetic characteristics of Cotoneaster multiflorus in Xinglong Mountain
Source: Front Plant Sci. 2025 Aug 5;16:1562491. doi: 10.3389/fpls.2025.1562491 (PMC12361200; doi:10.3389/fpls.2025.1562491)
Supplement: Supplementary file 1 [file DataSheet1.docx]

**Supplementary material**

**Influences of environmental and leaf functional traits variations on** **physiological characteristics of *Cotoneaster multiflorus* in Xinglong Mountain**

Xiaodong Ma^a^, Ling Han^b*^, Chengzhang Zhao^a,c*^, Dingyue Liu^a^

^a^ College of Geography and Environmental Science, Northwest Normal University, Lanzhou 730070, China

^b^ School of Art and Design, Lanzhou Jiaotong University, Lanzhou 730070, China

^c^ Xinglongshan Forest Ecosystem National Positioning Observation and Research Station, Lanzhou 730100, China.

*Corresponding author

[**hling0327@163.com**](mailto:hling0327@163.com)

**zhaocz601@163.com**

**TABLES**

**Table S1** Atmospheric environmental indicators for different slope directions.

| Factors | Slope | | | |
| --- | --- | --- | --- | --- |
|  | Southern slope | Eastern slope | Western slope | Northern slope |
| PAR  (μmol·m^–2^·s^–1^) | 1315.44 ± 123.49a | 1106.55 ± 20.51b | 1007.49 ± 53.55c | 927.10 ± 24.25d |
| TE (℃) | 29.65 ± 1.54a | 27.87 ± 0.96b | 27.11 ± 1.02b | 25.03 ± 1.0c |
| VPD (kPa) | 0.149 ± 0.002a | 0.130 ± 0.008b | 0.098 ± 0.008c | 0.089 ± 0.006d |

Different letters in each row represent significant differences (*P* < 0.05).

Significant differences were observed in PAR, TE, and saturated VPD across different slope directions (P < 0.05, Table 1). PAR, TE, and VPD exhibited a decreasing trend from the southern to the northern slope. The highest values were recorded on the southern slope, while the lowest were observed on the northern slope, reflecting reductions of 29.52%, 15.58%, and 40.27%, respectively.

**Table S2** Soil Environmental Elements for Different Slope Orientations. Different letters in each row represent significant differences (*P* <0.05).

| Factor | Slope | | | |
| --- | --- | --- | --- | --- |
|  | Southern Slope | Eastern slope | Western Slope | Northern Slope |
| SWC (%) | 24.09±1.61b | 27.70±2.08ab | 28.77±1.84a | 30.43±2.88a |
| ST (℃) | 22.09±1.35a | 21.80±1.84a | 18.37±1.48b | 16.09±1.07c |
| TN (mg·g^-1^) | 3.65±0.22c | 3.79±0.19c | 4.70±0.76b | 5.21±0.29a |
| SOC (mg·g^-1^) | 49.16±2.79b | 53.78±1.32ab | 54.82±1.43a | 55.72±3.18a |
| TP (mg·g^-1^) | 0.69±0.052bc | 0.74±0.078b | 0.88±0.057a | 0.90±0.022a |

Different letters in each row represent significant differences (*P* <0.05).

Significant differences were observed in SWC, ST, TN, SOC, and TP across different slope directions (P < 0.05, Table 2). As the slope direction shifted from south to north, SWC, TN, SOC, and TP increased by 26.32%, 42.74%, 13.34%, and 30.43%, respectively. In contrast, soil temperature exhibited a decreasing trend, declining by 27.16% from the southern to the northern slope.

**Table S3** The ANOVA was used to test aspect treatments on leaf morphology and photosynthetic fluorescence characteristics. The values of P < 0.05 are in bold.

| Dependent variable | Factors | Df | F | P |
| --- | --- | --- | --- | --- |
| Pn | Aspects | 3 | 59.315 | **<0.001** |
| Tr |  | 3 | 6.915 | **<0.001** |
| Ci |  | 3 | 77.612 | **<0.001** |
| Gs |  | 3 | 51.227 | **<0.001** |
| WUE |  | 3 | 95.091 | **<0.001** |
| NPQ |  | 3 | 54.062 | **<0.001** |
| qP |  | 3 | 138.024 | **<0.001** |
| ETR |  | 3 | 344.202 | **<0.001** |
| Y(II) |  | 3 | 49.366 | **<0.001** |
| fv/fm |  | 3 | 30.503 | **<0.001** |
| VLA |  | 3 | 24.022 | **<0.001** |
| VD |  | 3 | 29.644 | **<0.001** |
| LA |  | 3 | 26.733 | **<0.001** |
| LT |  | 3 | 15.319 | **<0.001** |
| SD |  | 3 | 51.523 | **<0.001** |

**Table S4** Variations of rETR under low and high light intensities on different slope aspects.

| PAR (μmol·m^-2^·s^-1^) | South | East | West | North |
| --- | --- | --- | --- | --- |
| <295 | 19.99 | 20.76 | 18.11 | 23.44 |
| 295~660 | 58.90 | 62.90 | 45.80 | 50.03 |
| >660 | 76.85 | 84.53 | 55.73 | 53.03 |

**Table S5** Analysis of variance results for Figure 7

| Name | Explains % | Contribution % | pseudo-F | P |
| --- | --- | --- | --- | --- |
| VD | 12.5 | 53.6 | 11.1 | 0.002 |
| SD | 8.3 | 35.5 | 8.0 | 0.004 |
| LA | 1.5 | 6.6 | 1.5 | 0.234 |
| VLA | 0.8 | 3.3 | 0.7 | 0.394 |
| LT | 0.2 | 1.0 | 0.2 | 0.792 |

**Table S6** Analysis of variance results for Figure 9

| Name | Explains % | Contribution % | pseudo-F | P |
| --- | --- | --- | --- | --- |
| SOC | 28.4 | 50.0 | 31.0 | 0.002 |
| TP | 16.2 | 28.6 | 22.6 | 0.002 |
| PAR | 3.5 | 6.1 | 5.1 | 0.022 |
| VPD | 4.7 | 8.3 | 7.5 | 0.004 |
| ST | 1.6 | 2.8 | 2.6 | 0.098 |
| SWC | 1.8 | 3.1 | 2.9 | 0.076 |
| TN | 0.6 | 1.1 | 1.0 | 0.336 |

**FIGS**


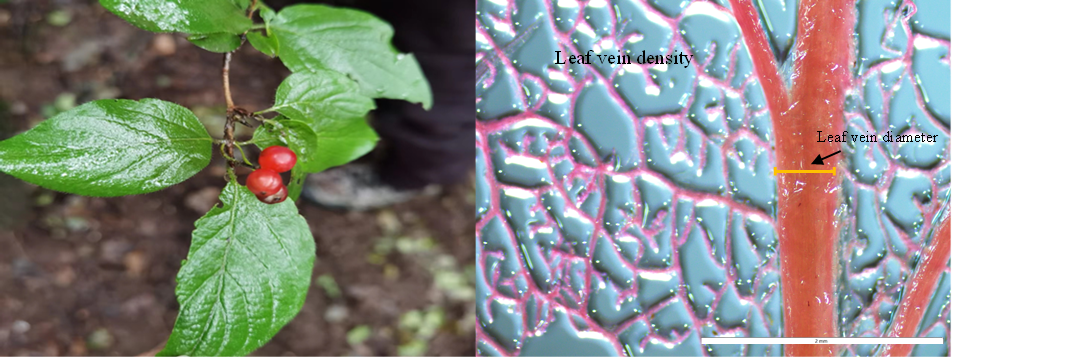


**Fig. S1** Electron microscopic image of *Cotoneaster multiflorus* leaf and vein traits


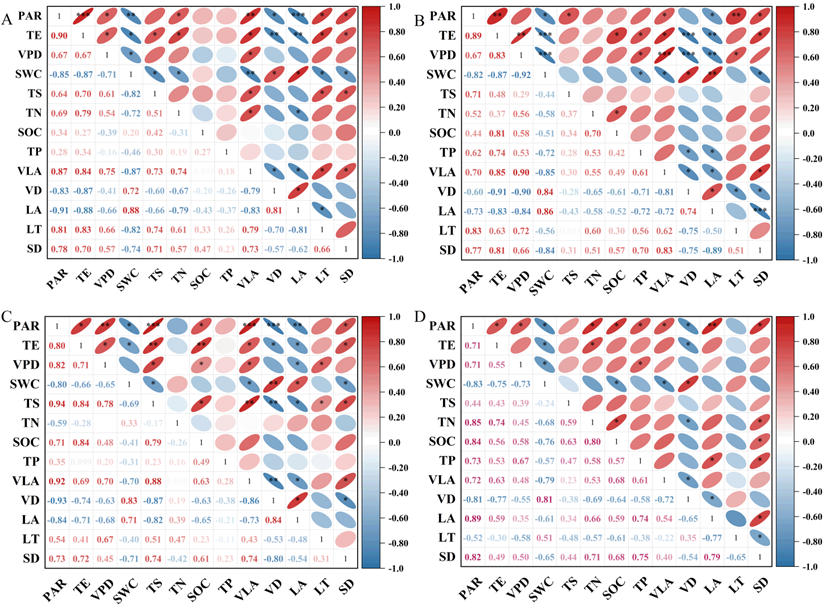


**Fig. S2** Pearson analysis of leaf vein traits of *Cotoneaster multiflorus* with environmental factors on different slope aspects. A, southern slope. B, eastern slope. C, western slope. D, northern slope. (*, P < 0.05; **, P < 0.01; ***, P < 0.001).

PAR, photosynthetically active radiation; TE, atmospheric temperature; VPD, saturated water-vapour pressure difference; SWC, soil water content; TS, soil temperature; TN, total nitrogen; SOC, soil organic carbon content; TP, total phosphorus；VLA, leaf vein density; VD, leaf vein diameter; LA, leaf area; LT, leaf thickness; SD, stomatal density.


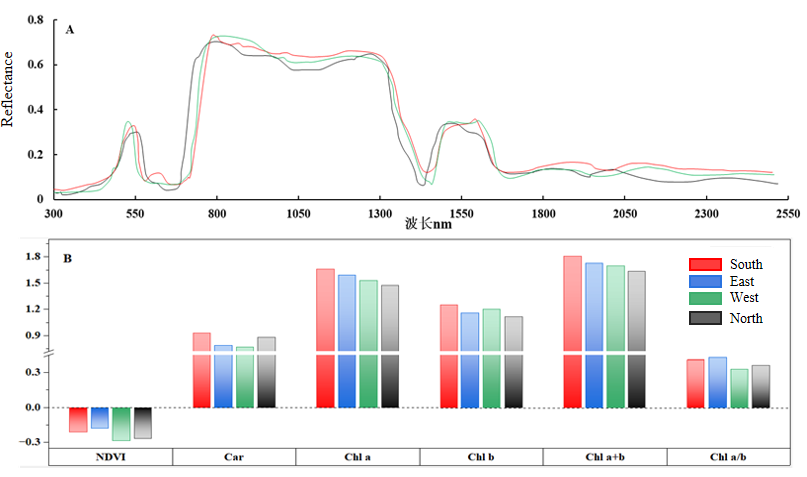


**Fig. S3**. Spectrogram (A) and chlorophyll content parameters (B) of *Cotoneaster multiflorus* leaves of different slope orientations. NDVI, normalised vegetation index; Car, carotenoid; Chl a, chlorophyll a; Chl a+b, chlorophyll a+b; Chl b, chlorophyll b content; Chl a/b, chlorophyll a/b.

According to leaf spectra of different slope directions, the chlorophyll absorption band in the visible band of 400-700 nm on the Northern Slope is significantly wider than that in other slope directions, indicating that the chlorophyll of leaves on the Northern Slope is more active, and the active trend of chlorophyll is north slope > East slope > South slope > West slope. Due to the strong absorption of chlorophyll in the 700-1300 nm red band and the high reflection in the infrared band, the reflectivity of this region rises rapidly, which is called the "red edge" of plant reflection. We found that the red edge of the north slope moves to the short wave, indicating that the chlorophyll content of the north slope is low, and the red edge of the south slope moves to the long wave direction, indicating that the chlorophyll content is high. The overall trend is south slope > East slope > West slope > North slope.

With the slope, direction changed from south to north, the normalized vegetation index NDVI showed an inverted U-shaped trend, with the maximum value (0.66) appearing on the eastern slope and the minimum value (0.52) appearing on the western slope, and the overall decrease was 21.21%. The variation of carotenoid (Car) was U-shaped, with the maximum value appearing on the south slope and the minimum value appearing on the west slope, and the decrease was 31.06%. Chlorophyll a (Chl a) and chlorophyll a+b (Chl a+b) showed a decreasing trend, and the maximum value appeared in the south slope, and the minimum value appeared in the north slope, and the decrease was 30.00 % and 27.78 %, respectively. The content of chlorophyll b (Chl b) decreased as a whole, with the maximum value (18 mg·g^-1^) on the south slope and the minimum value (13 mg·g^-1^) on the north slope, decreasing by 27.78%. Chlorophyll a/b (Chl a/b) showed an N-type trend, with the maximum value (2.69 mg·g^-1^) appearing on the eastern slope and the minimum value (2.13 mg·g^-1^) appearing on the western slope, with a decrease of 20.82%.
